# Point-of-Care Approaches for Meningitis Diagnosis in a Low-Resource Setting (Southwestern Uganda): Observational Cohort Study Protocol of the “PI-POC” Trial

**DOI:** 10.2196/21430

**Published:** 2020-11-04

**Authors:** Giulia Gaudenzi, Elias Kumbakumba, Reza Rasti, Deborah Nanjebe, Pedro Réu, Dan Nyehangane, Andreas Mårtensson, Milly Nassejje, Jens Karlsson, John Mzee, Peter Nilsson, Stephen Businge, Edmund Loh, Yap Boum II, Helene Andersson-Svahn, Jesper Gantelius, Juliet Mwanga-Amumpaire, Tobias Alfvén

**Affiliations:** 1 Department of Global Public Health Karolinska Institutet Stockholm Sweden; 2 Division of Nanobiotechnology Department of Protein Science KTH Royal Institute of Technology, SciLifeLab Stockholm Sweden; 3 Department of Paediatrics and Child Health Faculty of Medicine Mbarara University of Science and Technology Mbarara Uganda; 4 MSF Epicentre Mbarara Research Centre Mbarara Uganda; 5 Division of Affinity Proteomics Department of Protein Science KTH Royal Institute of Technology, SciLifeLab Stockholm Sweden; 6 Department of Women's and Children's Health, International Maternal and Child Health Uppsala University Uppsala Sweden; 7 Department of Microbiology, Tumor, and Cell Biology BioClinicum Karolinska University Hospital Stockholm Sweden; 8 Holy Innocents Children’s Hospital Mbarara Uganda; 9 SCELSE Nanyang Technological University Singapore Singapore

**Keywords:** global health, central nervous system infections, pediatrics, diagnostics, low-resource settings, meningitis, Uganda, children

## Abstract

**Background:**

A timely differential diagnostic is essential to identify the etiology of central nervous system (CNS) infections in children, in order to facilitate targeted treatment, manage patients, and improve clinical outcome.

**Objective:**

The Pediatric Infection-Point-of-Care (PI-POC) trial is investigating novel methods to improve and strengthen the differential diagnostics of suspected childhood CNS infections in low-income health systems such as those in Southwestern Uganda. This will be achieved by evaluating (1) a novel DNA-based diagnostic assay for CNS infections, (2) a commercially available multiplex PCR-based meningitis/encephalitis (ME) panel for clinical use in a facility-limited laboratory setting, (3) proteomics profiling of blood from children with severe CNS infection as compared to outpatient controls with fever yet not severely ill, and (4) Myxovirus resistance protein A (MxA) as a biomarker in blood for viral CNS infection. Further changes in the etiology of childhood CNS infections after the introduction of the pneumococcal conjugate vaccine against *Streptococcus pneumoniae* will be investigated. In addition, the carriage and invasive rate of *Neisseria meningitidis* will be recorded and serotyped, and the expression of its major virulence factor (polysaccharide capsule) will be investigated.

**Methods:**

The PI-POC trial is a prospective observational study of children including newborns up to 12 years of age with clinical features of CNS infection, and age-/sex-matched outpatient controls with fever yet not severely ill. Participants are recruited at 2 Pediatric clinics in Mbarara, Uganda. Cerebrospinal fluid (for cases only), blood, and nasopharyngeal (NP) swabs (for both cases and controls) sampled at both clinics are analyzed at the Epicentre Research Laboratory through gold-standard methods for CNS infection diagnosis (microscopy, biochemistry, and culture) and a commercially available ME panel for multiplex PCR analyses of the cerebrospinal fluid. An additional blood sample from cases is collected on day 3 after admission. After initial clinical analyses in Mbarara, samples will be transported to Stockholm, Sweden for (1) validation analyses of a novel nucleic acid–based POC test, (2) biomarker research, and (3) serotyping and molecular characterization of *S. pneumoniae* and *N. meningitidis*.

**Results:**

A pilot study was performed from January to April 2019. The PI-POC trial enrollment of patients begun in April 2019 and will continue until September 2020, to include up to 300 cases and controls. Preliminary results from the PI-POC study are expected by the end of 2020.

**Conclusions:**

The findings from the PI-POC study can potentially facilitate rapid etiological diagnosis of CNS infections in low-resource settings and allow for novel methods for determination of the severity of CNS infection in such environment.

**Trial Registration:**

ClinicalTrials.gov NCT03900091; https://clinicaltrials.gov/ct2/show/NCT03900091

**International Registered Report Identifier (IRRID):**

DERR1-10.2196/21430

## Introduction

### Pediatric Central Nervous System Infections and Their Etiology

Infections of the central nervous system (CNS) are life-threatening conditions affecting thousands of children worldwide, resulting in significant morbidity and mortality, and contributing to the current burden of disease in low-income countries [1,2]. When correctly treated, mortality falls significantly. However, many survivors are often left with neurological sequelae [3-5]. In most African countries, the etiology of CNS infections among children is often unknown due to the lack of advanced laboratory tests [6,7]. Although CNS infections can be caused by different infective agents, such as bacteria, viruses, and parasites, the symptoms of the infection, such as fever, are similar [8,9]. The common symptoms of CNS infection make it difficult to distinguish between different causes of infection clinically, posing challenges to clinical fever case management. Globally, the most common pathogens causing bacterial CNS infections beyond the neonatal period are *Neisseria meningitidis, Streptococcus pneumoniae*, and *Haemophilus influenzae* type B (Hib) [10]. When managing bacterial infections, it is important and sometimes crucial that antibiotics, if available, are administered without delay [11]. At the same time, the irrational use of antibiotics for the treatment of nonbacterial infections is a global public health concern that leads to increased development of antibiotic resistance [12]. In order to minimize preventable deaths and severe outcomes in children, it is crucial to direct research toward better and robust diagnosis of CNS infections.

### Etiology of Childhood CNS Infections in Southwestern Uganda

Uganda is a low-income country with a high prevalence of malaria, HIV, and other infectious diseases. In the Mbarara district of Southwestern Uganda, a child presenting with fever at a health facility is typically suspected of having either malaria or an unidentified bacterial/viral infection. Such patients are routinely tested for malaria using antigen-based rapid diagnostic point-of-care (POC) tests, commonly known as malaria rapid diagnostic tests (RDTs); if the test result is positive, the patient is treated for malaria. However, if the test result is negative, then empiric antibiotics are often prescribed without confirmation of the bacterial cause of infection [12]. One of the major reasons for this routine is the lack of adequate diagnostic methods [13]. A study conducted in the Mbarara district in Uganda from 2009 to 2012 showed that the bacterial etiologies of childhood CNS infection to primarily be *S. pneumoniae*, nontyphoid Salmonella, and Hib [14]. Vaccination against Hib and *S. pneumoniae*, 2 major causative bacterial agents in childhood CNS infection, has been added to the child immunization program in Uganda in 2003 and 2013, respectively. As described elsewhere, immunization programs have changed etiologies of invasive infectious diseases, allowing for previously infrequent pathogens to rise and take leading roles in infection etiology [15]. Whether this epidemiological shift is also happening in Uganda is important to study.

### A Need for Novel Rapid Microbiological POC Tests Integrated Into the Laboratory

#### Development of POC tests for CNS infections

The current gold-standard laboratory methods to confirm suspected cases of CNS infection are bacterial culture and PCR analyses of cerebrospinal fluid (CSF) and blood [16]. However, a vast number of cases in sub-Saharan Africa occur in areas with little or no access to laboratory diagnostics, resulting in mistreatment and poor clinical outcomes [6]. To improve clinical management of patients with suspected CNS infections in resource-limited settings, new field-friendly, easy-to-use, low-cost, and reliable diagnostic POC assays are greatly needed. To maximize the POC benefit, we have previously shown that design and development of tests must be conducted with a focus on needs and capacities of intended end users [17].

#### Multi-POC for Bacterial Meningitis Developed for Resource-Limited Settings

We have currently developed protein-, immuno-, and DNA-based platforms amenable for the POC multiplex format [18,19]. The DNA technologies are paper based, using printed microarrays to capture specific DNA targets by vertical flow and displaying the results colorimetrically [20]. These methods make use of traditional PCR as well as recombinase polymerase amplification, a nucleic acid amplification technique which does not require thermal cycling. Recombinase polymerase amplification combines the advantage of sensitivity and short turnaround time for analyses, making it well suited for laboratories without capacity for performing advanced molecular-based assays [21]. Paper-based POC tools offer not only a superior advantage in terms of costs and simplicity of use, but also when it comes to logistics, as they are easy to transport, store, and ultimately dispose safely via incineration.

#### A Commercially Available Multiplex PCR Instrument for Meningitis/Encephalitis Diagnostics

Concurrently, a new PCR meningitis/encephalitis (ME) panel is being marketed as a multiplex and fast instrument for integrated analysis of CSF specimens [22]. An increasing number of clinics in high-income countries (HICs) are now implementing its use as it has shown good performance for the diagnosis of CNS infections [23]; however, no studies have so far, to our knowledge, evaluated its clinical performance, influence on patient management, or clinical usability for the diagnosis of pediatric CNS infections in low-income settings.

#### Novel Biomarkers for Future POCs

A promising biomarker related to viral infections is Myxovirus resistance protein A (MxA). MxA is an intracellular protein that is upregulated upon activation of the antiviral defense. Because of its potential to function as a biomarker for viral infective agents, MxA is possibly an important complement to current inflammatory biomarkers that mostly focus on bacterial infection [24-26]. The correlation between MxA and the presence of viral encephalitis has previously been reported; however, this is only through histological analyses of brain tissue samples [27].

The mapping of the proteomic characteristics of febrile children with varying clinical signs of severe disease using an established, highly multiplexed antibody suspension bead protein microarray platform (Luminex) will be carried out [28,29]. Multivariate and machine-learning methods can be employed for the extraction of protein expression profiles that robustly correlate or predict clinical patterns such as disease severity, hospitalization, organ failure, and other complications.

#### Molecular Mechanisms of Potential Virulence Traits

The pathogen *N. meningitidis* is known to cause rapid onset of severe sepsis and meningitis. Several virulence factors are well known but how the pathogen progresses from an asymptomatic colonizer to an invasive infection remains unclear. Previous research has identified a specific capsule RNA thermosensor that is able to sense environmental temperature [30]. This temperature-sensing mechanism enables the bacterium to increase capsule production to evade host complement-mediated killing [31]. Specific sequence polymorphisms of the RNA thermosensor have also been linked to invasive *N. meningitidis* isolates [32]. Identification and surveillance of these RNA elements within naturally occurring populations of *N. meningitis* could aid the further development of tailored diagnostics and treatment.

### Aim

The overarching aim of the Pediatric Infection-Point-of-Care (PI-POC) clinical trial is to strengthen the capacity for differential diagnosis and management of childhood CNS infections in low-income health systems.

The specific objectives of the trial are the following (Table 1):

• To evaluate the diagnostic accuracy of novel nucleic acid-based assays for etiological diagnosis of bacterial CNS infection.

• To evaluate clinical performance, influence on patient management, and clinical usability of a commercially available multiplex PCR-based ME panel in managing pediatric CNS infection in a low-income setting.

• To compare differences in the etiology of confirmed childhood CNS infection before and after the introduction of the pneumococcal conjugate vaccine.

• To study the putative biomarker MxA in plasma in cases with viral CNS infection in comparison with that in cases of nonviral CNS infection.

• To study the proteomic profile, in CSF and in plasma, of children with severe versus uncomplicated infections.

• To study the prevalence, serogroup distribution, and molecular characteristics of *N. meningitidis* virulence traits.

**Table 1:** Summary of objectives, samples, and aims of the PI-POC^a^ study.

Objective	Aim	Specimen analyses	Specimen utilized
I	Validating a novel nucleic acid-based multi-POC^b^ method developed for the identification of CSF^c^ pathogens.	Sweden	CSF and cases
II	Evaluating the clinical utilization of a commercially available multiplex ME^d^ PCR panel in a low-income setting.	Uganda	CSF and cases
III	Updating current etiology of pediatric CNS^e^ infections in Mbarara and comparing it to the pre-pneumococcal conjugate vaccine era.	Uganda/Sweden	CSF and cases
IV	Validating MxA^f^ as a blood biomarker for differentiation of viral/nonviral cause of CNS infection.	Sweden	Plasma and cases and controls
V	Comparing proteomic characteristics of severe/nonsevere childhood infection and identification of novel biomarkers for severe pediatric infections.	Sweden	CSF, cases, plasma, and cases and controls
VI	Identifying mechanisms of virulence traits of *Neisseria meningitidis* isolated from pediatric patients.	Sweden	Nasopharyngeal swabs and cases and controls

^a^PI-POC: Pediatric Infection-Point-of-Care (trial).

^b^POC: point of care.

^c^CSF: cerebrospinal fluid.

^d^ME: meningitis/encephalitis.

^e^CNS: central nervous system.

^f^MxA: Myxovirus resistance protein A.

## Methods

### Study Site and Design

The PI-POC trial is a prospective observational study of children aged 0 months to 12 years presenting with symptoms of CNS infection. It also includes outpatient controls with a fever that are clinically assessed as not severely ill. The study is being conducted at the Pediatric Departments of Mbarara Regional Referral Hospital (MRRH) and Holy Innocents Children’s Hospital (HICH), Mbarara District, Uganda. MRRH is the main health facility and regional referral hospital in the southwestern region of Uganda and even receives patients from Tanzania, Rwanda, and the Democratic Republic of Congo. It is a teaching hospital for the medical faculty of the Mbarara University of Science and Technology (MUST) and has an admission capacity of 460 beds, outpatient and inpatient services, and a pediatric 70-bed ward that admits about 5000 children annually, mainly newborns and children with infectious diseases. HICH is a private pediatric nonprofit hospital established in 2009 and has a capacity of 60 beds. It offers inpatient and outpatient services, basic clinical laboratory (biosafety level 1), and counseling services. The Epicentre Laboratory is a biosafety level 3 facility, located within the campus of MUST and adjacent to MRRH. It includes parasitology, mycobacteriology, microbiology, and molecular biology (PCR, real-time qPCR, GeneXpert, BioFire FilmArray) laboratories and has biochemistry, hematology, and serology capacities. The laboratory is currently in the process of undergoing quality accreditation and operates on the principles of Good Clinical Laboratory Practice. Biobanking of samples in liquid nitrogen, –80°C and –20°C, is available on-site. Shipment of samples is conducted according to International Air Transport Association regulations.

Recruitment of patients commenced in January 2019 and was to end in June 2020. However, due to the current COVID-19 pandemic, the country underwent a total lockdown from March to June 2020, during which the study activity was paused. The study resumed mid-June and will continue until September 2020. The trial is registered at ClinicalTrials.gov (registration number NCT03900091).

### Study Participants

Children aged 0 months to 12 years who meet the case or control definition criteria, and for whom informed consent was obtained from the parent or guardian were recruited at the pediatric wards of both MRRH and HICH by the attending medical officer.

#### Case Definition

Children are suspected to have a CNS infection if they have fever or a history of fever in the past 48 hours (except for children younger than 9 months who may present with fever, normal body temperature, or hypothermia) and recent onset of any of the following at inclusion:

- Nontraumatic reduced level of consciousness (in preverbal children, this corresponds to Blantyre coma score <4 for those aged <9 months, and <5 for those older than 9 months (up to 12 years of age); in verbal children, this corresponds to Glasgow Coma Scale score <15);
- Prostration, hypotonia/hypertonia, unexplained irritability;
- Severe headache (severe enough to require hospitalization);
- Photophobia;
- Neck stiffness or bulging fontanel;
- Prolonged, partial, or multiple seizure(s);
- Focal neurological signs;
- In children older than 18 months: Kernig sign (flexion of the hip 90° with subsequent pain in legs extension) or Brudzinski sign (involuntary flexion of the knees and hips after passive flexion of the neck);
- Skin petechiae; and
- Cheyne–Stokes breathing.

Cases are recruited from the emergency department and inpatient ward of HICH and MRRH, when clinical presentation instigates suspicion of CNS infection and when the aforesaid inclusion criteria have been met. However, due to the often-ambiguous presentation of CNS infection symptoms, attending medical officers may include suspected cases even if the stated criteria are not met. The inclusion criteria of those children will be clearly mentioned in the case report form (CRF), and inclusion has to be validated by the principal investigator (EK) in Mbarara.

#### Control Definition

Controls included children aged 0 months to 12 years with a fever and not meeting the case definition, seeking care at the outpatient departments of MRRH or HICH. For every case, 1 sex- and age-matched control is included. When age matching, we allow flexibility to match up to 1 month of age for newborns, within 6 months for infants, and within a year for children from 1 to 12 years. The control definition is chosen to cater for all stated objectives of the trial, without subjecting healthy children to unnecessary and often painful diagnostic procedures.

### Biological Samples

Biosamples are collected from participants of the study, preferably before the initiation of antibiotic therapy (Table 2). In any instance, emergency care for the patients has priority over study procedures and sample collection.

#### Cerebrospinal Fluid (Cases Only)

CSF is collected through lumbar puncture (LP) by study clinicians at MRRH or HICH. LP is delayed or not performed in the presence of contraindications (signs of elevated intracranial pressure, focal neurological signs, local infection in the area of the puncture, signs of bleeding disorders, and cardiorespiratory compromise). The collected CSF is prioritized for patient care and routine primary laboratory diagnostics at the Epicentre Research Laboratory. Residual CSF is biobanked for further analyses.

To control for possible contamination, a sample of sterile water was poured into test tubes that are used for CSF collection. This was done at regular intervals during the trial, following the same procedure as when CSF is being collected from patients.

#### Blood (Cases and Controls)

##### Cases

Up to 6 mL of venous blood is collected from cases and prioritized for routine primary diagnostics upon study inclusion. Any residue is put aside for study purposes. On day 3 of the hospital stay, an additional study sample of 1-2 mL of blood is collected and cryopreserved for the longitudinal studies.

##### Controls

Capillary or venous samples are collected for routine primary diagnostics, and additionally, up to 1 mL is sampled and cryopreserved for study purposes.

#### Nasopharyngeal Swab (Cases and Controls)

One NP swab is collected from each suspected case or control. Swabs are frozen in a medium containing skim milk, tryptone, glucose, and glycerin and will be sent to the Karolinska Institutet.

### Microbiological and Biochemical Analyses

All clinical analyses are performed on-site at the Epicentre Research Laboratory. Primary clinical analyses of CSF, such as microscopy, cytology, Gram staining (when white blood cell count is 10 cells/mm^3^ or if sample is hematic), microbiology culture, and biochemistry assay (glucose, protein, lactate) are performed. One aliquot of 200 µL of CSF is used for the multiplex ME panel (BioFire FilmArray ME Panel; BioFire Diagnostics) according to the manufacturer protocol. Blood analyses include Malaria RDT (SD Bioline Malaria Ag Pf/Pan RDT; Standard Diagnostics), HIV RDT using a sequential algorithm of Determine HIV 1/2 (Alere), followed by HIV 1/2 STAT-PAK (Chembio) for reactive samples, and SD Bioline HIV-1/2 (version 3.0; Standard Diagnostics) as a tie-breaker for discordant results, according to Uganda’s national guidelines. Other analyses included blood culture (BD BACTEC Peds Plus; Beckton Dickinson & Company), hematology (XN-550, Sysmex), biochemistry (Cobas C111; Roche), and malaria microscopy if RDT is positive. NP swabs are cryopreserved at –80°C for whole-genome sequencing and typing of relevant strains; such analyses will be conducted at the Science for Life Laboratory (SciLifeLab) and Karolinska Institutet.

**Table 2:** Summary of samples and analyses in Uganda and Sweden.^a^

Specimens (type and tests)		Inclusion (Day 0)	Day 3	Clinical analyses (when/where)	Type of research analyses (when/where)
**Cases**					
	Blood: Hematology, biochemistry, HIV serology (and confirmation), malaria RDT^b^, culture, plasma biobank	6 mL	1-2 mL	Routine clinical analyses (same day at the Epicentre Laboratory).	Proteomics analyses for profiling of biomarkers including MxA^e^ (later at the SciLifeLab^f^).
	CSF^c^: Biochemistry, microscopy and cytology, culture, ME^d^-PCR panel, PCR, biobank	2 mL	—	Routine clinical analyses including ME-PCR panel (same day at the Epicentre Laboratory).	Proteomics analyses for profiling of biomarkers including MxA (later at the SciLifeLab).
	Nasopharyngeal swabs: Biobank	1 swab	—	—	Typing of bacteria and bacterial whole-genome sequencing (later at the Karolinska Institutet and SciLifeLab).
**Controls**					
	Blood: Hematology, HIV serology (and confirmation), malaria RDT, biobank	1-2 mL	—	Routine clinical analyses (same day at the Epicentre Laboratory).	Proteomics analyses for profiling of biomarkers including MxA (later at the SciLifeLab).
	Nasopharyngeal swabs: Biobank	1 swab	—	—	Typing of bacteria and bacterial whole-genome sequencing (later at the Karolinska Institutet and SciLifeLab).

^a^Additional testing for clinical care, such as TB GeneXpert or TB ZN, was performed according to clinical needs and at the discretion of the medical officer.

^b^RDT: rapid diagnostic test

^c^CSF: cerebrospinal fluid.

^d^ME: meningitis/encephalitis.

^e^MxA: Myxovirus resistance protein A

^f^SciLifeLab: Science for Life Laboratory.

### Study Variables

A CRF for cases and controls is filled upon study enrollment. CRF includes demography, medical history, vaccination status, and use of anti-infective medicines prior to inclusion. The following information is registered by the study doctor recruiting cases: respiratory rate, consciousness according to the Glasgow Coma Scale, pulse, peripheral oxygen saturation, weight, body temperature, administered antipyretic medication (<4 hours), vomiting, neck stiffness, bulging fontanelle, unexplainable screaming, opisthotonos, central cyanosis, jaundice, inability to feed, and capillary refill time. Information regarding admission, length of stay, radiological routine clinical examination, microbiology and chemistry analyses, treatment, discharge diagnosis, and complications will be retrospectively collected from the medical records.

Laboratory data are collected by the biologist responsible for the study laboratory activities. Data entry and management are performed by the data management team in Mbarara. Deidentified data are double-entered in the REDCap (Research Electronic Data Capture) database, a web-based software for electronic capture of clinical research data [33].

### Study Size, Power Calculation, and Statistical Methods

With the primary aim of this exploratory study being the evaluation of new diagnostic assays in development, a proper power calculation cannot be done. In a previous study, 459 cases with suspected CNS infection were included [14]. In half of them, a causative pathogen was identified in the CSF. Based on previous reports, we can assume that the introduction of pneumococcal conjugate immunization to the Mbarara district has halved the incidence of bacterial CNS infections caused by *S. pneumoniae* [14]. However, other causative bacterial agents of CNS infection are unlikely to have decreased in the region. We thus believe that we should be able to include approximately 160 cases in which a causative pathogen can be identified if we include the same number of cases with suspected CNS infection as in the previous study. This means that around 300 cases with suspected CNS infection will be included in this study. These numbers will be adequate for the validation of the assays.

### Statistical Methods and Data Analysis

Standards for Reporting of Diagnostic Accuracy Studies (STARD) will be followed to report results [34]. For continuous variables, mean, median (IQR), SD, and maximum and minimum values will be given. Variables will be described by their percentages and CI. Clinically relevant differences in protein levels of samples versus controls will be reported using appropriate statistical methods in accordance with sample size, number, and distribution of the data. Data will be presented with 95% CI and a *P*-value of <.05 will be considered significant.

### Ethics Approval and Consent to Participate

The final protocol has received ethical approval from the Institutional Ethical Review Board of MUST in Uganda (ref. 22/05-18) and the Regional Ethical Review Board in Stockholm, Sweden (ref. 2018/1676-31/1). Approval for the study was granted by the Uganda National Council for Science and Technology (UNCST Reference No HS 2508). All substudies are conducted in accordance with the Helsinki Declaration and follow guidelines for Good Clinical Practice and Good Laboratory Practice. Guardians of potential participants will receive written information on the study and can decide freely whether or not to participate. Their choice to participate or not will not have any influence on the continued clinical management of their child. No identifying data are recorded in the database or used in the analysis. The children are identified by a numeric identifier. Documents including the name of the child, name of the guardian, inclusion number, and contact address are kept locked and accessible only to investigators for the purpose of active follow up. All procedures included in this study, except for the NP swabs, are standard procedures in the management of cases of CNS infections. Good clinical practice and good laboratory practice will be followed. This study might have some direct benefits for the patient, as in the standard routine practice only bacterial culture is performed on the CSF samples. The results from additional tests are communicated directly to the ward as soon as they are obtained, and the treatment is adapted based on these results.

## Results

The Regional Ethical Review Board in Stockholm approved the study (ref. 2018/1676-31/1) in September 2018. A pilot study was performed from January 2019 to April 2019, to evaluate the study protocol, the sample transfer logistics from the hospitals to the Epicentre Research Laboratory, and the turnover of patients with suspected CNS infections at both facilities. Helpful information about patient screening and recruitment procedures, including matching control patients, was retrieved during the pilot study. Improvements to the recruitment process, handling of logistics, and transfer of study data between Mbarara and Stockholm were made. We identified a cultural barrier and misconception for LP procedure in the local population, as seen in other sub-Saharan countries [35], and it was promptly addressed by the medical officers by providing additional counseling to the children’s guardians. Enrollments of the patients started in April 2019. The FilmArray instrument was installed at the Epicentre Research Laboratory in May 2019.

## Discussion

### Protocol Overview

CNS infections in children remain a leading cause of death and life-long disability. Thus, early diagnostics are playing a pivotal role in ensuring the right treatment is delivered in a timely manner. The findings from the PI-POC study aim to increase knowledge about CNS infection etiology in Southwestern Uganda using a nucleic acid–based assay amenable for POC use and an ME panel to evaluate the performance of this assay in a burdened health system. Further, it aims at describing the current epidemiological landscape of CNS infection in the area and whether any changes have occurred with the introduction of pneumococcal conjugate and Hib vaccines. Interestingly, not many cases of *N. meningitidis* have been reported in the area [14] although Mbarara is in the proximity of the African meningitis belt. Therefore, the PI-POC study aims at characterizing the serogroup, sequence type, and strain characteristics of all the identified cases of meningococcal meningitis. MxA is a putative blood biomarker that could discern viral and bacterial infection, and it will be investigated in this cohort via a massive proteomics profiling of CSF and plasma.

### POC-Amenable Solutions for CNS Infection Detection in a Low-Income Context

Unknown fever etiology in children is a barrier to successful treatment and rapid recovery [36,37]. Findings from the PI-POC study aim at improving near-patient differential diagnosis of CNS infection etiology using POC diagnostics for CSF infections. Because many of the cases occur in rural areas, where access to a physician and advanced diagnostics is limited, the PI-POC trial employs high-throughput affinity proteomics approaches to investigate plasma protein profiles of children having either bacterial or viral CNS infections. Such an approach could aid in the further development of novel POC diagnostics for CNS infection in blood.

### Limitations of the Study

Patients are recruited from 2 different hospital sites in the same area which might limit the findings to that region. Often, antibiotics are administered before LP; hence, most cases have negative results in microbiological culture. Another limitation of the study is the potential selection bias of patients during recruitment. According to the clinical picture, the medical officer may suspect a CNS infection even when not all criteria have been met. In such a scenario, cases are always validated/discussed with the principal investigator (EK) of the study in Mbarara and if included, described in detail in the CRF.

The Epicentre Research Laboratory is an advanced biosafety level 3 facility and might not be a representation of other diagnostic laboratories in Uganda. Finally, the ME multiplex PCR panel includes 14 different pathogens known to cause meningitis; however, this panel is not epidemiologically targeted to Uganda, and other agents, such as nontyphoid Salmonella, which are known to cause meningitis in children in the area [14], are not included in the panel.

### Requirements for Future RDTs in Low-to-Middle-Income Countries

POC tests should be designed with good knowledge of the health system in which they will be used. The mere availability of rapid or simple tests does not automatically ensure their adoption or scale-up [38]. Several barriers are preventing the successful uptake of POC testing. One barrier is usability; several tests are designed in high-income countries without incorporating the user’s perception into the process. Rapid tests designed for a context might fail in another. We successfully addressed this by first conducting a qualitative study and continuously interacting with the end users of our POC prototype [17]. Other cultural barriers might prevent the test from being used, such as the cultural acceptability of LP [35]. During the PI-POC trial pilot, it was evident that effort was needed by the physician to address the importance of LP to diagnose CNS infections. Logistics also plays a big part in the design of rapid POC tests and should incorporate knowledge of distribution (temperature-sensitive reagents might fail), usage, and finally of the disposal, as safe laboratory waste disposal is a pressing issue in many contexts. An economic effort is also needed to make sure tests have an advantageous package cost for low- to middle-income countries, as many tests remain too expansive for use outside high-income countries or special settings in low- to middle-income countries.

POC tests should not replace advanced laboratory facilities and should not be seen as an antagonist to the central laboratory; in fact, strong knowledge of clinical diagnostics is a requirement for successful POC testing in all settings [39]. As we have learnt from the current COVID-19 pandemic, diagnostic capacity plays a significant role in successful disease surveillance, detection, and response [40] and should not be overlooked by the global health community.

## Abbreviations

CNS: central nervous system
CRF: case report form
CSF: cerebrospinal fluid
Hib: Haemophilus influenzae type B
LP: lumbar puncture
ME: meningitis/encephalitis
MxA: Myxovirus resistance protein A
NP: nasopharyngeal
PI-POC: Pediatric Infection-Point-of-Care (trial)
POC: point of care
RDT: rapid diagnostic test

## References

[1] GBD 2016 Meningitis Collaborators (2018). Global, regional, and national burden of meningitis, 1990-2016: a systematic analysis for the Global Burden of Disease Study 2016. Lancet Neurol.

[2] Furyk J, Swann O, Molyneux E (2011). Systematic review: neonatal meningitis in the developing world. Trop Med Int Health.

[3] Edmond K, Clark A, Korczak VS, Sanderson C, Griffiths UK, Rudan I (2010). Global and regional risk of disabling sequelae from bacterial meningitis: a systematic review and meta-analysis. Lancet Infect Dis.

[4] Ramakrishnan M, Ulland A, Steinhardt L, Moïsi JC, Were F, Levine O (2009). Sequelae due to bacterial meningitis among African children: a systematic literature review. BMC Med.

[5] Schmidt H, Heimann B, Djukic M, Mazurek C, Fels C, Wallesch C, Nau R (2006). Neuropsychological sequelae of bacterial and viral meningitis. Brain.

[6] Petti CA, Polage CR, Quinn TC, Ronald AR, Sande MA (2006). Laboratory medicine in Africa: a barrier to effective health care. Clin Infect Dis.

[7] Huttunen P, Lappalainen M, Salo E, Lönnqvist T, Jokela P, Hyypiä T, Peltola H (2009). Differential diagnosis of acute central nervous system infections in children using modern microbiological methods. Acta Paediatr.

[8] Yoshizato R, Koga H (2020). Comparison of initial and final diagnoses in children with acute febrile illness: A retrospective, descriptive study: Initial and final diagnoses in children with acute fever. J Infect Chemother.

[9] Barbi E, Marzuillo P, Neri E, Naviglio S, Krauss BS (2017). Fever in Children: Pearls and Pitfalls. Children (Basel).

[10] Oordt-Speets A, Bolijn R, van Hoorn RC, Bhavsar A, Kyaw M (2018). Global etiology of bacterial meningitis: A systematic review and meta-analysis. PLoS One.

[11] Tacon C, Flower O (2012). Diagnosis and management of bacterial meningitis in the paediatric population: a review. Emerg Med Int.

[12] Kemigisha E, Nanjebe D, Boum Y, Langendorf C, Aberrane S, Nyehangane D, Nackers F, Mueller Y, Charrel R, Murphy RA, Page A, Mwanga-Amumpaire J (2018). Antimicrobial treatment practices among Ugandan children with suspicion of central nervous system infection. PLoS One.

[13] Caliendo AM, Gilbert DN, Ginocchio CC, Hanson KE, May L, Quinn TC, Tenover FC, Alland D, Blaschke AJ, Bonomo RA, Carroll KC, Ferraro MJ, Hirschhorn LR, Joseph WP, Karchmer T, MacIntyre AT, Reller LB, Jackson AF, Infectious Diseases Society of America (IDSA) (2013). Better tests, better care: improved diagnostics for infectious diseases. Clin Infect Dis.

[14] Page A, Boum Ii Yap, Kemigisha E, Salez N, Nanjebe D, Langendorf C, Aberrane S, Nyehangane D, Nackers F, Baron E, Charrel R, Mwanga-Amumpaire J (2017). Aetiology and Outcomes of Suspected Infections of the Central Nervous System in Children in Mbarara, Uganda. Sci Rep.

[15] Adams W (1993). Decline of Childhood Haemophilus influenzae Type b (Hib) Disease in the Hib Vaccine Era. JAMA.

[16] He T, Kaplan S, Kamboj M, Tang Y (2016). Laboratory Diagnosis of Central Nervous System Infection. Curr Infect Dis Rep.

[17] Rasti R, Nanjebe D, Karlström J, Muchunguzi C, Mwanga-Amumpaire J, Gantelius J, Mårtensson A, Rivas L, Galban F, Reuterswärd P, Andersson Svahn H, Alvesson HM, Boum Y, Alfvén T (2017). Health care workers' perceptions of point-of-care testing in a low-income country-A qualitative study in Southwestern Uganda. PLoS One.

[18] Nybond S, Réu P, Rhedin S, Svedberg G, Alfvén T, Gantelius J, Svahn HA (2019). Adenoviral detection by recombinase polymerase amplification and vertical flow paper microarray. Anal Bioanal Chem.

[19] Reuterswärd P, Gantelius J, Andersson Svahn H (2015). An 8 minute colorimetric paper-based reverse phase vertical flow serum microarray for screening of hyper IgE syndrome. Analyst.

[20] Dias JT, Svedberg G, Nystrand M, Andersson-Svahn H, Gantelius J (2017). Rapid signal enhancement method for nanoprobe-based biosensing. Sci Rep.

[21] James A, Macdonald J (2015). Recombinase polymerase amplification: Emergence as a critical molecular technology for rapid, low-resource diagnostics. Expert Rev Mol Diagn.

[22] Poritz MA, Blaschke AJ, Byington CL, Meyers L, Nilsson K, Jones DE, Thatcher SA, Robbins T, Lingenfelter B, Amiott E, Herbener A, Daly J, Dobrowolski SF, Teng DH, Ririe KM (2011). FilmArray, an automated nested multiplex PCR system for multi-pathogen detection: development and application to respiratory tract infection. PLoS One.

[23] Soucek DK, Dumkow LE, VanLangen KM, Jameson AP (2019). Cost Justification of the BioFire FilmArray Meningitis/Encephalitis Panel Versus Standard of Care for Diagnosing Meningitis in a Community Hospital. J Pharm Pract.

[24] Forster J, Schweizer M, Schumacher R, Kaufmehl K, Lob S (1996). MxA protein in infants and children with respiratory tract infection. Acta Paediatr.

[25] Engelmann I, Dubos F, Lobert P, Houssin C, Degas V, Sardet A, Decoster A, Dewilde A, Martinot A, Hober D (2015). Diagnosis of viral infections using myxovirus resistance protein A (MxA). Pediatrics.

[26] Nakabayashi M, Adachi Y, Itazawa T, Okabe Y, Kanegane H, Kawamura M, Tomita A, Miyawaki T (2006). MxA-based recognition of viral illness in febrile children by a whole blood assay. Pediatr Res.

[27] Lampe J, Schneider-Schaulies S, Aguzzi A (2003). Expression of the interferon-induced MxA protein in viral encephalitis. Neuropathol Appl Neurobiol.

[28] Bachmann J, Burté F, Pramana S, Conte I, Brown BJ, Orimadegun AE, Ajetunmobi WA, Afolabi NK, Akinkunmi F, Omokhodion S, Akinbami FO, Shokunbi WA, Kampf C, Pawitan Y, Uhlén M, Sodeinde O, Schwenk JM, Wahlgren M, Fernandez-Reyes D, Nilsson P (2014). Affinity proteomics reveals elevated muscle proteins in plasma of children with cerebral malaria. PLoS Pathog.

[29] Reuterswärd P, Bergström S, Orikiiriza J, Lindquist E, Bergström S, Andersson Svahn H, Ayoglu B, Uhlén M, Wahlgren M, Normark J, Ribacke U, Nilsson P (2018). Levels of human proteins in plasma associated with acute paediatric malaria. Malar J.

[30] Loh E, Lavender H, Tan F, Tracy A, Tang CM (2016). Thermoregulation of Meningococcal fHbp, an Important Virulence Factor and Vaccine Antigen, Is Mediated by Anti-ribosomal Binding Site Sequences in the Open Reading Frame. PLoS Pathog.

[31] Loh E, Kugelberg E, Tracy A, Zhang Q, Gollan B, Ewles H, Chalmers R, Pelicic V, Tang CM (2013). Temperature triggers immune evasion by Neisseria meningitidis. Nature.

[32] Karlsson J, Eichner H, Andersson C, Jacobsson S, Loh E (2020). Identification and characterisation of novel hyper-capsulation RNA thermosensor variants in Neisseria meningitidis and their association with invasive meningococcal disease: a genetic and phenotypical investigation and molecular epidemiological study. The Lancet Microbe (Forthcoming).

[33] Harris PA, Taylor R, Thielke R, Payne J, Gonzalez N, Conde JG (2009). Research electronic data capture (REDCap)--a metadata-driven methodology and workflow process for providing translational research informatics support. J Biomed Inform.

[34] Cohen JF, Korevaar DA, Altman DG, Bruns DE, Gatsonis CA, Hooft L, Irwig L, Levine D, Reitsma JB, de Vet HCW, Bossuyt PMM (2016). STARD 2015 guidelines for reporting diagnostic accuracy studies: explanation and elaboration. BMJ Open.

[35] Thakur K, Mateyo K, Hachaambwa L, Kayamba V, Mallewa M, Mallewa J, Nwazor E, Lawal T, Mallum C, Atadzhanov M, Boulware D, Birbeck G, Siddiqi O (2015). Lumbar puncture refusal in sub-Saharan Africa: A call for further understanding and intervention. Neurology.

[36] Iroh Tam P, Obaro SK, Storch G (2016). Challenges in the Etiology and Diagnosis of Acute Febrile Illness in Children in Low- and Middle-Income Countries. J Pediatric Infect Dis Soc.

[37] Johansson E, Kitutu F, Mayora C, Awor P, Peterson S, Wamani H, Hildenwall H (2016). It could be viral but you don't know, you have not diagnosed it: health worker challenges in managing non-malaria paediatric fevers in the low transmission area of Mbarara District, Uganda. Malar J.

[38] Pai N, Vadnais C, Denkinger C, Engel N, Pai M (2012). Point-of-care testing for infectious diseases: diversity, complexity, and barriers in low- and middle-income countries. PLoS Med.

[39] Pai M, Walia K, Boehme C (2019). Essential medicines and essential diagnostics: a package deal. Lancet Public Health.

[40] COVID-19 Clinical Research Coalition. Electronic address: nick.white@covid19crc.org (2020). Global coalition to accelerate COVID-19 clinical research in resource-limited settings. Lancet.

